# Vascular adhesion protein-1–targeted [^68^Ga]Ga-DOTA-Siglec-9 PET/CT in Takayasu arteritis: imaging vascular inflammation and treatment reponse

**DOI:** 10.1093/rap/rkag076

**Published:** 2026-07-07

**Authors:** Simon M Petzinna, Jim Küppers, Daniel Ginzburg, Markus Essler, Valentin S Schäfer

**Affiliations:** Medical Clinic and Polyclinic III Internal Medicine with a focus on Oncology, Hematology, and Rheumatology, University Hospital Bonn, University Bonn, Bonn, Germany; Clinic and Polyclinic for Nuclear Medicine, University Hospital Bonn, University Bonn, Bonn, Germany; Clinic and Polyclinic for Nuclear Medicine, University Hospital Bonn, University Bonn, Bonn, Germany; Clinic and Polyclinic for Nuclear Medicine, University Hospital Bonn, University Bonn, Bonn, Germany; Medical Clinic and Polyclinic III Internal Medicine with a focus on Oncology, Hematology, and Rheumatology, University Hospital Bonn, University Bonn, Bonn, Germany

Key message • Imaging with [^68^Ga]Ga-DOTA-Siglec-9 PET/CT may enable detection of inflammation-related vascular tracer uptake in Takayasu arteritis and support monitoring of treatment-associated changes.

Dear Editor, Takayasu arteritis (TAK) is an autoimmune vasculitis that primarily affects the aorta and its major branches, predominantly in young women aged 20–30 years. Imaging plays a pivotal role in diagnosis, and the current ACR/European Alliance of Associations for Rheumatology (EULAR) classification criteria mandate morphological evidence of vasculitis [1]. Diagnosis often relies on MRI or [^18^F]fluorodeoxyglucose ([^18^F]FDG) PET/CT. The latter enables visualization of metabolic activity in inflamed vessel walls. However, residual tracer uptake is frequently observed even during clinical remission, potentially reflecting vascular remodelling or residual disease activity [2]. Therefore, according to EULAR recommendations [1], [^18^F]FDG-PET/CT serves as a second-line modality after MRI in TAK, emphasizing the need for more inflammation-specific tracers.

Recently, [^68^Ga]Ga-DOTA-Siglec-9 has been introduced as a novel PET tracer targeting vascular adhesion protein-1 (VAP-1), which is expressed on endothelial and vascular smooth muscle cells [3, 4]. Under inflammatory conditions (e.g. TNF-α, IFN-γ, IL-1β), VAP-1 translocates to the cell surface, mediating leucocyte adhesion and transmigration through interaction with its ligand, sialic acid-binding immunoglobulin-like lectin (Siglec)-9, on neutrophils and monocytes [3, 4]. A soluble form (sVAP-1) is released through MMP-dependent cleavage of its membrane-bound counterpart [3, 4]. The (s)VAP-1/Siglec-9 axis contributes both to enzyme-independent immune cell recruitment and to enzyme-dependent oxidative deamination, amplifying vascular injury and inflammation [3, 4]. Our group previously demonstrated increased [^68^Ga]Ga-DOTA-Siglec-9 uptake in patients with relapsing GCA [5, 6] and idiopathic inflammatory myopathy [7]. Given the overlap between GCA and TAK, exploring [^68^Ga]Ga-DOTA-Siglec-9-PET/CT in TAK is of particular interest.

This report describes the first exploratory and hypothesis-generating application of this tracer in two patients with TAK. The first patient, a 34-year-old woman, was diagnosed in 2015 (blood pressure discrepancy of >15 mmHg and constitutional symptoms) and presented in June 2025. The initial [^18^F]FDG-PET/CT (2015) had demonstrated inflammation of the carotid arteries, aortic arch and descending aorta. Serial MRI between 2018 and 2020 revealed progressive aneurysmatic dilation of the ascending aorta (37–44 mm). A follow-up [^18^F]FDG-PET/CT in August 2024 showed persistent low-grade uptake in the ascending aorta and right carotid artery.

The patient had been treated with subcutaneous tocilizumab since 2016, along with several unsuccessful tapering attempts of prednisolone. At presentation, she was receiving 3 mg prednisolone and reported mild night sweats. Comprehensive vascular ultrasound (GE LOGIQ E10, 2021) of the axillary, brachial, subclavian and carotid arteries [8] demonstrated increased intima-media thickness (IMT) across multiple arteries (axillary: 1.1, 1.4 mm; left/right; carotid: 1.6, 2.6 mm; left/right; subclavian: 1.4, 1.4 mm; left/right). Laboratory results were within normal limits (Supplementary Table S1). Disease activity was classified as active (independent of PET findings) based on an integrated clinical assessment by the treating rheumatologist, including persistent symptoms and disease course.

The patient underwent [^68^Ga]Ga-DOTA-Siglec-9-PET/CT following intravenous administration of 200 MBq of tracer (radiosynthesis protocol: Supplementary Data S1). The injection was well tolerated. Image analysis, which was performed blinded to the final clinical assessment, revealed focal tracer uptake in the right carotid artery, while other ultrasound-identified arteries with IMT thickening showed no relevant accumulation (Fig. 1A). Longitudinal follow-up imaging was performed 4 months after the initial scan following renewed glucocorticoid exposure (20 mg/day with tapering over 3 months). In this examination (Fig. 1B), the previously observed focal tracer uptake in the right carotid artery was no longer detectable. This loss of signal paralleled clinical improvement, including resolution of night sweats.

**Figure 1:**
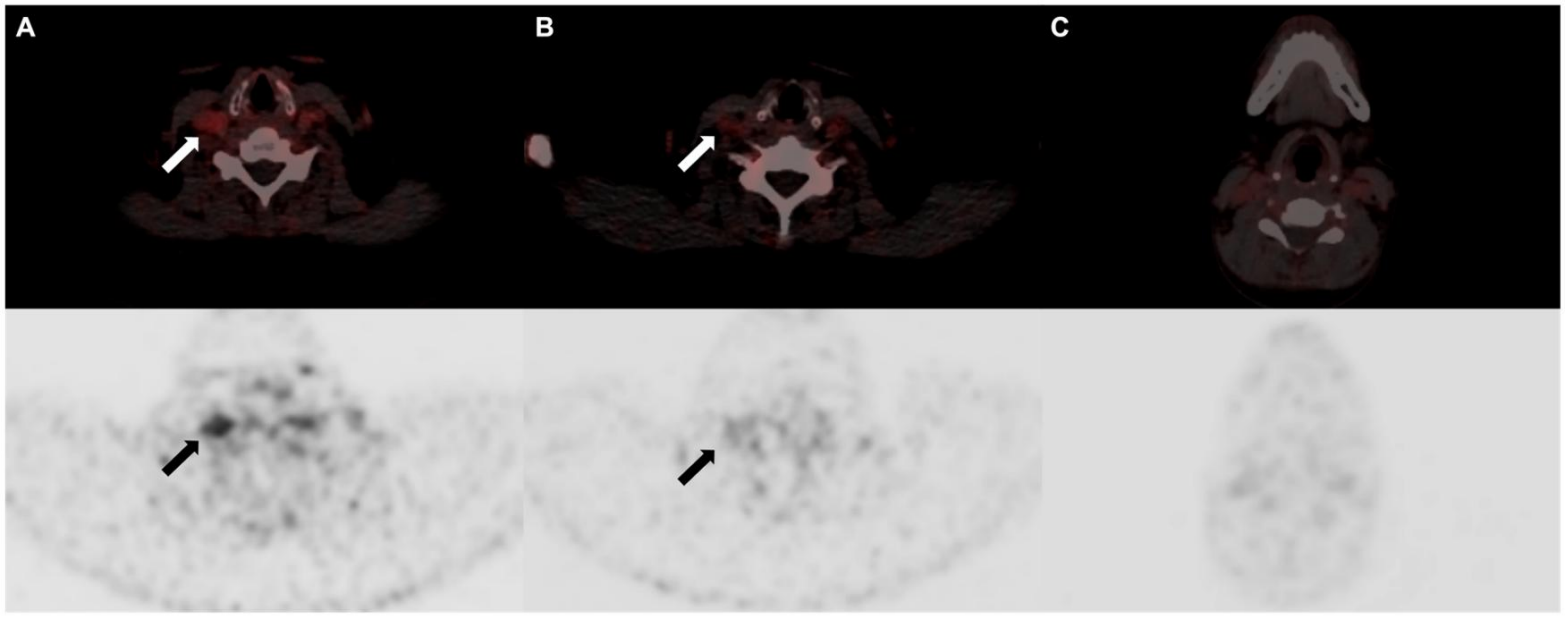
[^68^Ga]Ga-DOTA-Siglec-9 PET/CT images illustrating active TAK with longitudinal resolution of tracer uptake and a patient in remission. Representative axial PET/CT images demonstrating vascular tracer uptake in (A) the first patient with clinically active TAK, (B) the same patient at 4-month follow-up after additional prednisolone therapy and (C) the second patient with no active vasculitis. The upper panels show fused PET/CT images, while the lower panels display the corresponding PET images. Arrows indicate focal tracer uptake in the right carotid artery at baseline (A), with resolution of the signal on follow-up (B). No pathological vascular tracer uptake is observed in the second patient (C), demonstrating physiological tracer distribution. In the fused images, PET signal intensity is displayed using a standardized colour scale, where brighter colours correspond to higher [^68^Ga]Ga-DOTA-Siglec-9 uptake. The lower PET-only images are shown in grayscale, where darker signal intensity reflects higher tracer accumulation. Abbreviations: CT, computed tomography; PET, positron emission tomography; TAK, Takayasu arteritis

To provide context, images were compared with those obtained from a second TAK patient, who underwent [^68^Ga]Ga-DOTA-Siglec-9-PET/CT under identical imaging protocol due to initial clinical suspicion of relapse. The 25-year-old woman had been diagnosed in 2020 and was receiving tocilizumab since February 2024, combined with prednisolone (5 mg/day). At imaging, she reported mild night sweats. Vascular ultrasound demonstrated increased IMT of the carotid (3.8, 2.2 mm; right/left), axillary (0.9, 1.5 mm; right/left) and subclavian arteries (1.1, 1.2 mm; right/left). No laboratory abnormalities were detected (Supplementary Table S1). Within days, the patient showed spontaneous resolution of symptoms without escalation of therapy, leading to clinical dismissal of the initial suspicion of active disease. Correspondingly, image analysis revealed no focal or segmental tracer uptake (Fig. 1C).

Taken together, our study provides preliminary evidence that [^68^Ga]Ga-DOTA-Siglec-9-PET/CT may detect inflammation-related vascular tracer uptake in TAK, potentially reflecting local upregulation of VAP-1 within the inflamed vessel wall. The absence of focal tracer uptake in a clinically inactive case, its independence from residual structural IMT changes in non-active vascular segments and its resolution on longitudinal follow-up support an inflammation-related interpretation and potential sensitivity to changes in disease activity.

Targeting VAP-1 may therefore allow localization and characterization of active vascular inflammation and support disease monitoring. However, given the exploratory design and small sample size, these findings should be interpreted as hypothesis-generating rather than confirmatory. The close anatomical proximity of cervical arterial and venous structures, the spatial resolution limits of PET/CT and the lack of independent objective imaging correlates of disease activity preclude definitive attribution of tracer uptake to active vascular inflammation. Larger studies, including comparative imaging with [^18^F]FDG-PET/CT and MRI, are needed to define the diagnostic and prognostic value and to further elucidate the role of the VAP-1/Siglec-9 axis and the potential modulatory effects of IL-6 receptor blockade.

## Supplementary Material

rkag076_Supplementary_Data

## Data Availability

Data are available upon reasonable request from the corresponding author.
