# Supplementary material for: Vascular adhesion protein-1–targeted [68Ga]Ga-DOTA-Siglec-9 PET/CT in Takayasu arteritis: imaging vascular inflammation and treatment reponse
Source: Rheumatol Adv Pract. 2026 Jul 7;10(3):rkag076. doi: 10.1093/rap/rkag076 (PMC13373243; doi:10.1093/rap/rkag076)
Supplement: rkag076_Supplementary_Data [file rkag076_supplementary_data.zip › 25-291 Supplementary Data S1.docx]

*Supplementary Data S1. Radiochemical synthesis of [^68^Ga]Ga-DOTA-Siglec-9*

Gallium-68 was obtained from a germanium-68/gallium-68 generator (EZAG, Berlin, Germany). Synthesis was performed utilizing an automated cassette module (GAIA; Elysia‑Raytest, Straubenhardt, Germany). Standard fluidic and reagent kit for gallium-68 radiolabeling of peptides (ABX advanced biochemical compounds GmbH, Radeberg, Germany) were employed. TraceSelect water and ethanol Ph. Eur. were purchased from Merck (Darmstadt, Germany).

DOTA-Siglec-9 was obtained from ABX and diluted with TraceSelect water to achieve a final concentration of 1 mg/mL. For the synthesis, the module was equipped with 150.00 ± 5.00 µg DOTA-Siglec-9, 500 µL eluent, 2.49 mL ammonium acetate buffer (pH 4.5) and 200 µL ethanol. After radiolabeling at 65 ± 5 °C for 15.00 min, the reaction mixture was purified through C-18 solid phase extraction and the final product was obtained in isotonic sodium chloride solution.

For quality control, an aliquot of about 50 µL was taken from the final formulation before measurement of radioactivity. All chemicals were pure or analytical grade and used as received, unless otherwise specified.

Radioactivity of the final product was measured with a dose calibrator (ISOMED 2010, MED Nuklear-Medizintechnik Dresden GmbH, Dresden, Germany). Radiochemical purity was determined using glass microfiber chromatography paper impregnated with silica-gel (iTLC‑SG, Agilent Technologies, Santa Clara, USA) and analyzed with a single trace radioTLC‑scanner (PET-miniGita, Elysia-Raytest, Straubenhardt, Germany) and evaluation software (Gina Star TLC, Elysia-Raytest, Straubenhardt, Germany). iTLC-strips were developed in 0.1 M citric buffer (pH 4; Merck, Darmstadt, Germany) and 1 M ammonium acetate/methanol (1:1) (v:v). Furthermore, radioHPLC was used to determine the radiochemical purity and identification of the product species. RadioHPLC was performed utilizing Agilent 1260 Infinity II reverse phase HPLC system (Agilent Technologies, Santa Clara, USA) equipped with Gabi γ-HPLC flow detector (Elysia-Raytest, Straubenhardt, Germany) and a PC interface running Gina Star (Elysia-Raytest, Straubenhardt, Germany). A Nucleodur 100-3 C18 ec 125/4 column (Macherey-Nagel GmbH & Co. KG, Düren, Germany) was applied. The gradient utilized mobile phase A (deionized water + 0.1% TFA) and mobile phase B (acetonitrile + 0.1% TFA) at a flow rate of 0.7 mL/min starting with 100% A / 0% B to 0% A / 100% B within 20 min. pH was measured using pH-indicator strips MColorpHast 2.0−9.0 (Merck, Darmstadt, Germany). The approximate half-life of gallium-68 was determined using a dose calibrator (ISOMED 2010, MED Nuklear-Medizintechnik Dresden GmbH, Dresden, Germany). The nuclide specific energy of gallium-68 as well as germanium-68 breakthrough (48h post labeling) were measured using a multi‑channel‑analyzer for γ‑spectroscopy (MUCHA; Elysia-Raytest, Straubenhardt, Germany). Clear appearance was checked visually. Filter integrity was tested with GAIA (Elysia-Raytest, Straubenhardt, Germany).

Non-decay corrected (AY) as well as decay corrected radiochemical yield were calculated based on the activity trapped on the SCX, activity trapped on C-18 and remaining activity on C-18 after final formulation as measured by the module. Volume activity and apparent molar activity were calculated based on activity of the final product.

Radiosynthesis of [^68^Ga]Ga-DOTA-Siglec-9 with 1.91 ± 0.41 GBq gallium-68 revealed a decay corrected radiochemical yield of 77.7 ± 5.90% (63.9 ± 4.85% AY). A volume activity of 124 ± 7.00 MBq/mL and an apparent molar activity of 19.1 ± 1.05 MBq/nmol were obtained. Radiochemical purity was >98%.
