# Supplementary material for: Vascular adhesion protein-1–targeted [68Ga]Ga-DOTA-Siglec-9 PET/CT in Takayasu arteritis: imaging vascular inflammation and treatment reponse
Source: Rheumatol Adv Pract. 2026 Jul 7;10(3):rkag076. doi: 10.1093/rap/rkag076 (PMC13373243; doi:10.1093/rap/rkag076)
Supplement: rkag076_Supplementary_Data [file rkag076_supplementary_data.zip › 25-291 Supplementary Table.docx]

**Supplementary Table S1**: Clinical, laboratory, and imaging characteristics of two patients with Takayasu arteritis undergoing [⁶⁸Ga]Ga-DOTA-Siglec-9-PET/CT.

| **Parameter** | **Patient A (TAK, Relapse)** | **Patient B (TAK, Remission)** |
| --- | --- | --- |
| **Age (years)** | 34.4 | 25.8 |
| **Sex** | Female | Female |
| **Diagnosis (year)** | 2015 | 2020 |
| **Symptom onset** | 2011 | 2020 |
| **Clinical symptoms (at imaging)** | Night sweats, blood pressure discrepancy | Night sweats |
| **Treatment**  **(at imaging)** | Tocilizumab (since 2016),  Prednisolone 3 mg daily | Tocilizumab (since Feb 2024),  Prednisolone 5 mg daily |
| **CRP (mg/L)** | 0.6 | 2.4 |
| **Leucocytes (×10⁹/L)** | 7.9 | 12.6 |
| **Hemoglobin (g/dL)** | 12.0 | 12.4 |
| **Platelets (×10⁹/L)** | 287 | 251 |
| **Axillarian artery IMT (mm)** | 1.1 (left)/ 1.4 (right) | 1.5 (left)/ 0.9 (right) |
| **Subclavian artery IMT (mm)** | 1.3 (left)/ 1.4 (right) | 1.2 (left)/ 1.1 (right) |
| **Carotidal artery (mm)** | 2.6 (left)/ 1.6 (right) | 2.2 (left)/ 3.8 (right) |

*The table summarizes demographic data, treatment status, inflammatory markers, and IMT measurements in a case of active disease (Patient A) and remission (Patient B). Abbrv.: IMT: Intima Media Thickness, TAK: Takayasu Arteritis.*
